# Particle-Tracking Proton Computed Tomography-Data Acquisition, Preprocessing, and Preconditioning

**DOI:** 10.1109/access.2021.3057760

**Published:** 2021-02-08

**Authors:** BLAKE SCHULTZE, PANIZ KARBASI, CHRISTINA SAROSIEK, GEORGE COUTRAKON, CAESAR E. ORDOÑEZ, NICHOLAS T. KARONIS, KIRK L. DUFFIN, VLADIMIR A. BASHKIROV, ROBERT P. JOHNSON, KEITH E. SCHUBERT, REINHARD W. SCHULTE

**Affiliations:** 1 Department of Electrical and Computer Engineering, Baylor University, Waco, TX 76798, USA; 2 Bioinformatics Department, University of Texas—Southwestern, Dallas, TX 75390, USA; 3 Department of Physics, Northern Illinois University, DeKalb, IL 60115, USA; 4 Department of Computer Science, Northern Illinois University, DeKalb, IL 60115, USA; 5 Data Science and Learning Division, Argonne National Laboratory, Argonne, IL 60439, USA; 6 Department of Basic Sciences, Loma Linda University, Loma Linda, CA 92350, USA; 7 Department of Physics, University of California at Santa Cruz, Santa Cruz, CA 95064, USA

**Keywords:** Proton computed tomography, data acquisition, preprocessing, initial image formation

## Abstract

Proton CT (pCT) is a promising new imaging technique that can reconstruct relative stopping power (RSP) more accurately than x-ray CT in each cubic millimeter voxel of the patient. This, in turn, will result in better proton range accuracy and, therefore, smaller planned tumor volumes (PTV). The hardware description and some reconstructed images have previously been reported. In a series of two contributions, we focus on presenting the software algorithms that convert pCT detector data to the final reconstructed pCT images for application in proton treatment planning. There were several options on how to accomplish this, and we will describe our solutions at each stage of the data processing chain. In the first paper of this series, we present the data acquisition with the pCT tracking and energy-range detectors and how the data are preprocessed, including the conversion to the well-formatted track information from tracking data and water-equivalent path length from the data of a calibrated multi-stage energy-range detector. These preprocessed data are then used for the initial image formation with an FDK cone-beam CT algorithm. The output of data acquisition, preprocessing, and FDK reconstruction is presented along with illustrative imaging results for two phantoms, including a pediatric head phantom. The second paper in this series will demonstrate the use of iterative solvers in conjunction with the superiorization methodology to further improve the images resulting from the upfront FDK image reconstruction and the implementation of these algorithms on a hybrid CPU/GPU computer cluster.

## I. INTRODUCTION

The interest in the technological development of proton computed tomography, called pCT herein, has increased in recent years due to its potential to reduce the range uncertainty problem in proton therapy [1], [2]. The range of proton beams in a given patient is associated with substantial uncertainties, including the conversion of Hounsfield units (HU) to relative stopping power (RSP) with respect to water, daily variation in the patient setup as well as in the distribution and composition of tissues. These uncertainties are typically larger than those due to beam delivery and dose calculation, especially when the latter is performed with Monte Carlo algorithms. A common practice has been to add an additional margin of 3.5% plus 1 mm to the nominal range of a proton beam [3], which often makes the uncertainty margins between clinical target volume and planning target volume larger than those in photon therapy.

The application of pCT to proton treatment planning and pretreatment verification may substantially reduce the uncertainty margins in proton therapy because uncertainties in the conversion of x-ray CT HU to proton RSP are avoided. Proton CT will be very useful for detecting changes in anatomy and RSP distribution before treatment on a weekly or even daily basis for adaptive proton therapy.

There is currently no clinical pCT system in operation, but several groups worldwide have been working on technological solutions and have created preclinical prototypes. These vary from approaches to integrate the effect of penetrating proton pencil beams with existing dosimetry equipment to approaches employing sophisticated particle-tracking and calorimetric techniques borrowed from high-energy physics. See a recent review of these approaches in [4]. The tracking of single protons through the object generally leads to a lower dose and better image quality but has also been perceived as an impractical solution requiring complex equipment.

The purpose of this contribution is to present our approach to pCT data acquisition, preprocessing, and preconditioning for image reconstruction using single proton tracking and water-equivalent path length (WEPL) measurements to demonstrate that this solution is practical and clinically feasible. Our selection of suitable iterative image reconstruction algorithms will be presented in a forthcoming publication.

## II. HISTORICAL REVIEW OF pCT DEVELOPMENT

After physicist Robert Wilson pointed out the advantages of protons and heavier ions for radiation therapy [5], the first suggestion to use charged particles to obtain tomographic medical images as a second medical application was made by Allan Cormack in his now famous 1963 and 1964 publications on the concept of tomographic image reconstruction [6], [7]. For his work in tomographic image reconstruction methods, he was later awarded the Nobel Prize in Medicine of 1979. Cormack was less optimistic about the usefulness of protons for tomographic imaging compared to x-rays because he predicted that multiple Coulomb scattering on atomic nuclei would lead to rather blurry images. As published previously [8] and mentioned in this contribution and the companion paper on image reconstruction, the problem can be alleviated by estimating by estimating the most likely path (MLP) of individual particles based on tracking data. However, the resolution of proton CT remains inferior to that of x-ray CT.

About 10 years after Cormack’s initial suggestion, investigators at the Lawrence Berkeley National Laboratory tracked the positions of 900 MeV helium ions with a multi-wire proportional chamber and individually measured their residual range using a stack of plastic scintillators [9], thus realizing for the first time that the concept of a modern particle-tracking CT scanner was feasible. The data allowed the investigators to perform tomographic reconstructions that were then compared to those obtained with a commercial x-ray CT scanner. The authors emphasized that the dose for the single-particle-based helium CT scan was about up to 50 times lower than for the conventional x-ray CT scan.

In 1976, two investigators at the Harvard Cyclotron Lab, Allan Cormack and his colleague Andreas Koehler, demonstrated the first experimental pCT images [10]. The investigators measured the energy lost by 158 MeV protons traversing a 9.52 cm diameter, radially symmetric acrylic phantom. With this experiment, they demonstrated that density differences as small as 0.006 g/cm^3^ relative to the acrylic background density of 1.17 g/cm^3^ could be distinguished in the tomographic reconstruction. This experiment successfully demonstrated that protons could achieve density resolution superior to those attained in contemporary commercial x-ray scanners, which was about 1% at comparable imaging dose.

In the late 1970s and early 1980s, Kenneth Hanson and collaborators at the Los Alamos Meson Physics Facility (LAMPF) envisioned the first series of pCT experiments designed to bridge the gap between the physics laboratory and the clinic. Hanson’s first experimental system consisted of a hyper-pure germanium detector (HPGe) and a multi-wire proportional chamber (MWPC) to measure the residual energy and exit position of each proton, respectively. In a later implementation, a range telescope consisting of a stack of plastic scintillators was added. The suitability of these different systems, described in three landmark publications [11]–[13], were assessed to achieve minimum dose at the maximum possible count rate. In a later experiment with an upgraded readout system, human tissue specimens were scanned to demonstrate the clinical feasibility of pCT [14]. One important conclusion from these early pCT experiments was that measuring the exit position and direction of protons exiting the phantom would further improve spatial resolution. Another conclusion was that the dose advantage for pCT versus x-ray CT for ideal systems was about 4:1 for a 20 cm diameter phantom and 8:1 for a 30 cm diameter phantom.

The early exploration phase of proton and ion CT ended with the experiments at Los Alamos, and it took about 20 years before the next phase of development started. By that time, the clinical use of protons in hospital-based facilities was beginning to expand. Range uncertainties in proton therapy planning related to conversion of x-ray CT attenuation data to proton RSP were increasingly recognized as a challenge that needed to be addressed. In addition, there was a peculiar lack of image guidance technology in the proton treatment room that further aggravated the range uncertainty problem as tissues shifted or changed between treatments or were affected by internal organ motion during treatment. Proton CT was soon recognized as a potential remedy of the range uncertainty problem.

In early 2003, the U.S. pCT collaboration was formed during a 2-day meeting at Brookhaven National Laboratory, and plans were discussed to develop a pCT system using state-ofthe-art particle tracking and calorimeter detector technology. For image reconstruction, the collaboration decided to further develop the most likely path (MLP) concept, previously proposed by Schneider and Pedroni [15] using the particle tracking information [8] and combine this concept with iterative reconstruction techniques. In 2011, the pCT collaboration received funding from the National Institute of Biomedical Imaging and Bioengineering (NIBIB) to build a preclinical proton CT scanner that was able to track about 1 million protons per second and to acquire a complete 360-degree pCT acquisition of a head phantom in under 10 minutes. The collaboration was successful in this regard, as described in recent publications [16], [17].

Over the last 20 years, different approaches have been developed by various groups working in the field of proton (and ion) imaging for application in proton (and ion) therapy. For an overview of these approaches, the reader is referred to a recent review [4]. The general way in which proton imaging data are acquired can be divided into two conceptually different approaches: (a) The particle tracking (or list) mode, which uses technology similar to the one presented in this work and allows one to use the information from individual protons or ions traversing the patient and (b) the integration mode, which uses established therapeutic beam delivery modes of lower intensity and measures an integrated beam current dependent on the water-equivalent thickness (WET) of proton or ion beams traversing the patient, typically with existing dosimetric equipment. The integration mode is simpler and less expensive than the particle tracking mode, but comes at the cost of less resolution and, in particular, higher patient dose [4].

## III. pCT DETECTOR

The pCT detector, consisting of a tracker system and an energy detector, has been well documented in previous publications [16]–[18]. In this section, we provide a brief summary explaining the data collected from proton track histories for image reconstruction.

### A. TRACKER

The tracking planes measure the coordinates of each proton entering and exiting the phantom. The tracker consists of eight planes of silicon strip detectors. Each plane contains four individual detectors with vertical (*T*-plane) or horizontal (*V*-Plane) strip orientation and with small gaps (0.5 mm) between the individual detectors. The *T*-planes measure the *T* coordinates and the *V*-planes the *V* coordinates of each proton in two upstream and two downstream locations, respectively, allowing for the reconstruction of track segments. The four *T*- and *V*-plane pairs have an intra-pair distance of 5 mm and the inter-pair distance of 50 mm. The *T*-planes are always (by 5 mm) closer to the isocenter than the corresponding *V-*planes of each pair to allow for best coordinate measurement accuracy along the *T*-axis. Each tracker plane has an active area of 9 cm (vertically) by 36 cm (horizontally) with an rms spatial resolution of 70 *μ*m.

The (*T, V*) hit coordinates on the tracking planes are used to determine the entry and exit location as well as the entry and exit angle of protons incident on the phantom’s surface. The innermost tracking planes are separated by 30 cm to allow insertion of various phantoms. The angle *θ* of the rotational platform at each event is inferred from the time of its trigger and the constant 1 RPM rate of the platform.

### B. ENERGY DETECTOR

**FIGURE 1.**
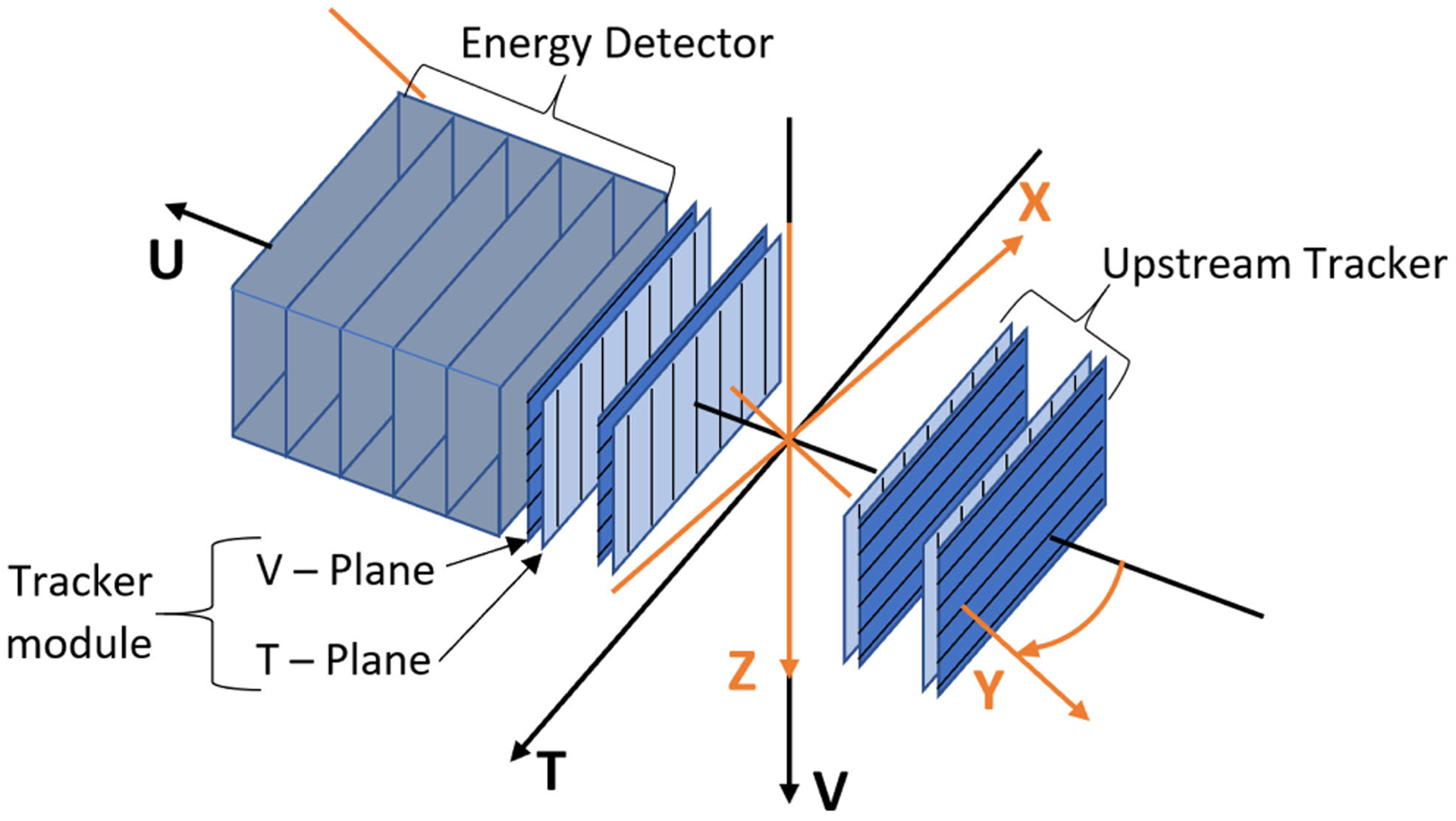
Proton CT coordinate systems (*T, U, V*) of the detector system and (*X, Y, Z*) of the object (not shown). The object rotates around the *V* axis during the data acquisition. There is an upstream and a downstream tracker, each consisting of two tracker modules. Each module consists of a pair of *T*- and *V*-planes. A uniform beam of 200 MeV protons enters the upstream tracker from the right and proceeds in the positive U direction. The rotation angle *θ* is the angle between the *Y* and *U* axes. *T*, *U*, and *V* form the basis for the isocentric tracking coordinates of each recorded track. After passing the tracker system, protons enter and are stopped in the energy detector, which consists of five scintillators stacked along the *U* axis.

The residual energy, *E*_*f*_, of each proton exiting the last tracker plane is measured with a five-stage plastic scintillator with photomultiplier tube (PMT) readout, as seen on the left in Fig. 1. The signal from the readout is converted to WEPL of the proton through the phantom using the calibration procedure described in Section VI. The scintillators also provide a trigger for data acquisition. A threshold is applied to the signal of the first three stages, which allow a trigger when any of the three scintillators give a signal above threshold. Each of the five scintillator stages has the same WET of 52.9 mm along the beam direction. Therefore, a pulse height measurement in the most distal stage registering the signal from the stopping proton can be used to infer the WEPL of the proton through the phantom.

The WEPL of each proton traversing the phantom is an important quantity in pCT needed for image reconstruction. Formally, it is related to the incident energy, *E*_*in*_, and the exit energy, *E*_*out*_, via the path integral:

(1) $$\text{WEPL}=\int_{E_{in}}^{E_{out}}\frac{dE}{{\frac{dE}{dx}}_{\text{water}}}$$

In principle, one could calculate the WEPL of individual protons by numerical integration; however, this is not practical because the energies of the incoming and outgoing proton traversing the phantom are not easy to derive from its initial energy *E*_0_ = 200 MeV and its measured final energy *E*_*f*_. Also, the mean excitation energy *I*_*water*_, a parameter in the Bethe formula for stopping power, is relatively uncertain. Therefore, we implemented a calibration procedure for each of the five stages of the energy detector that relates the signal in the stage where the proton stops to a known WEPL value through the calibration procedure, as described in Section VI.

As we will discuss in more detail in Section VII-D, WEPL is the fundamental quantity required to reconstruct the RSP along the estimated path of each proton. The relationship between WEPL for each proton path through a phantom and the RSP along its path is given by the path integral

(2) $$\text{WEPL}=\int_{\text{path}}RSP_{\text{path}}dl$$

where

(3) $$\text{RSP}=\frac{{\frac{dE}{dx}}_{\text{medium}}}{{\frac{dE}{dx}}_{\text{water}}}$$

These equations form the basis of the filtered back projection reconstruction described in Section VII-D.

## IV. LOW INTENSITY BEAM DELIVERY FOR pCT

**FIGURE 2.**
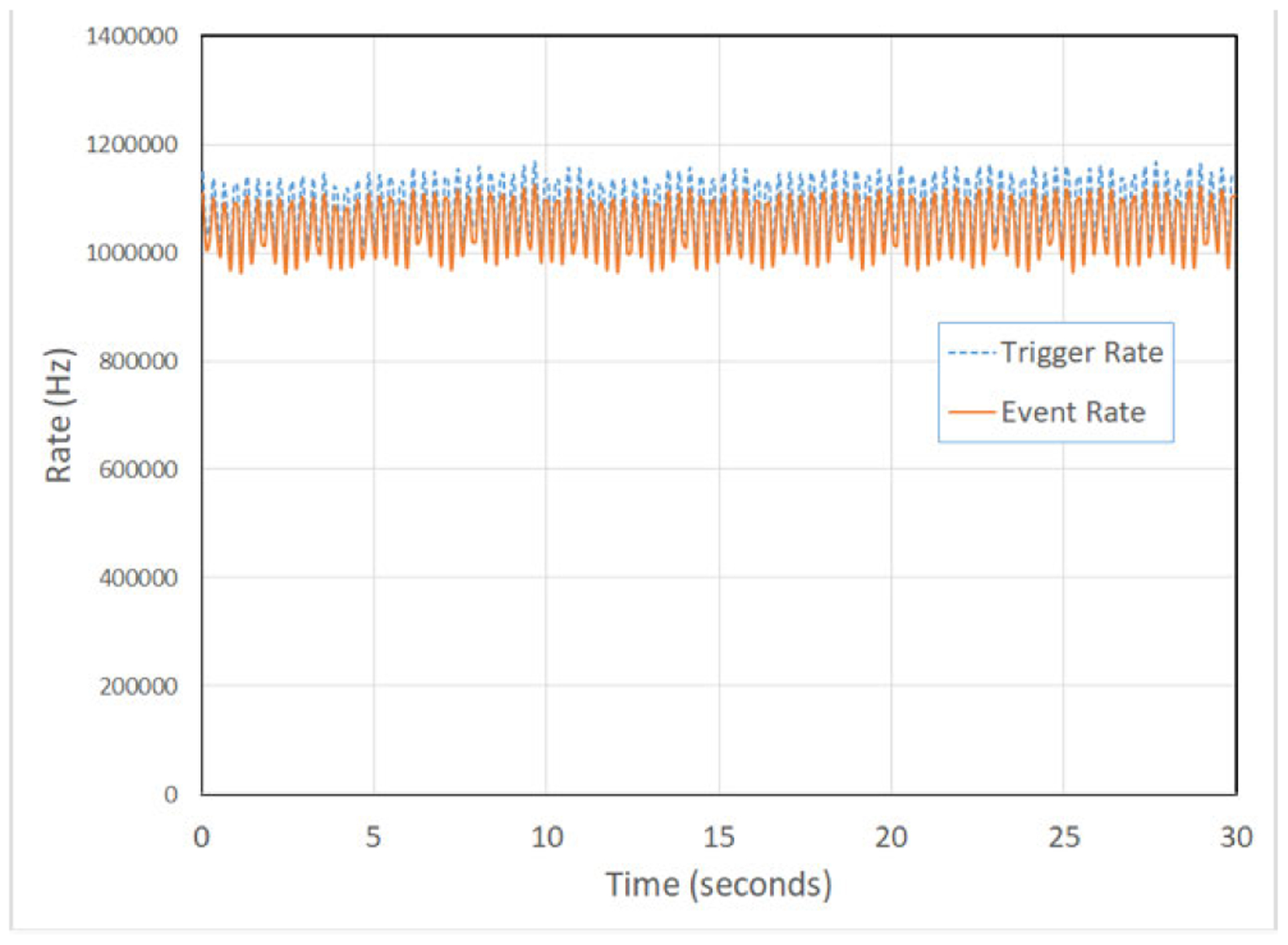
The time structure of the beam intensity (trigger rate and recorded event rate) is shown over a time interval of 30 seconds. Both rates are sampled approximately every 100 ms. Also, the recorded event rate is about 5% lower than the trigger rate due to dead time in the DAQ system.

The pCT detector has been tested on a horizontal clinical beam line at Northwestern Medicine Chicago Proton Center (NMCPC), which operates a 235 MeV IBA cyclotron. Protons of 200 MeV are delivered to the pCT scanner and phantom using a clinical beam delivery system with (*T, V*) directional wobbling. The beam spot size for these exposures is 4 cm full width at half maximum and the field size was 10 cm vertically by 30 cm horizontally. This produces a beam geometry of rectangular pyramid shape for imaging phantoms up to 25 cm diameter. With this beam delivery system, the effective source point related to magnetic bending in the horizontal direction is 230 cm from isocenter and the effective source point related to the magnetic bending in the vertical direction is 190 cm from isocenter. This effectively creates a divergent beam delivery system with two source points for the two directions of beam deflection. The sweep speed of the scanning magnets for a single proton field of 10 cm × 30 cm size is 3 Hz while the detector area is 9 cm × 36 cm. The beam intensity has been lowered for pCT operation so that the trigger rate remains near 1 MHz, as shown in Figure 2. A procedure was established to lower the intensity by a factor of 10^4^ below clinical treatment intensity. First, the intensity from the source is lowered to 2 nA for the extracted intensity from the IBA cyclotron just upstream of the Energy Selection System (ESS), which contains a polycarbonate energy degrader. Next, the collimators in the ESS that control the longitudinal and transverse emittance are partially closed so that about 10^6^ protons per second pass through the energy degrader. The intensity from the cyclotron is uniform to within ±2% percent and stable over the 6 minutes of the scan. The system is thus capable of acquiring data at a 1 MHz event rate with minimal dead time.

## V. ENERGY CALIBRATION AND CONVERSION TO WEPL

Reconstruction of the WEPL for each proton from the five-stage scintillator data relies on a calibration procedure to convert scintillator pulse height to MeV and then MeV to WEPL [19]. The original calibration procedure for pCT was described by [20]. Initially, *T* and *V* variations in light collection for each of the five scintillators are measured using several million 200 MeV protons accumulated with no phantom in place. The data are binned into 1 cm (*T, V*) intervals in each stage to generate correction factors that are used during the preprocessing to flatten the response profile across the 9 × 36 cm sensitive area.

**FIGURE 3.**
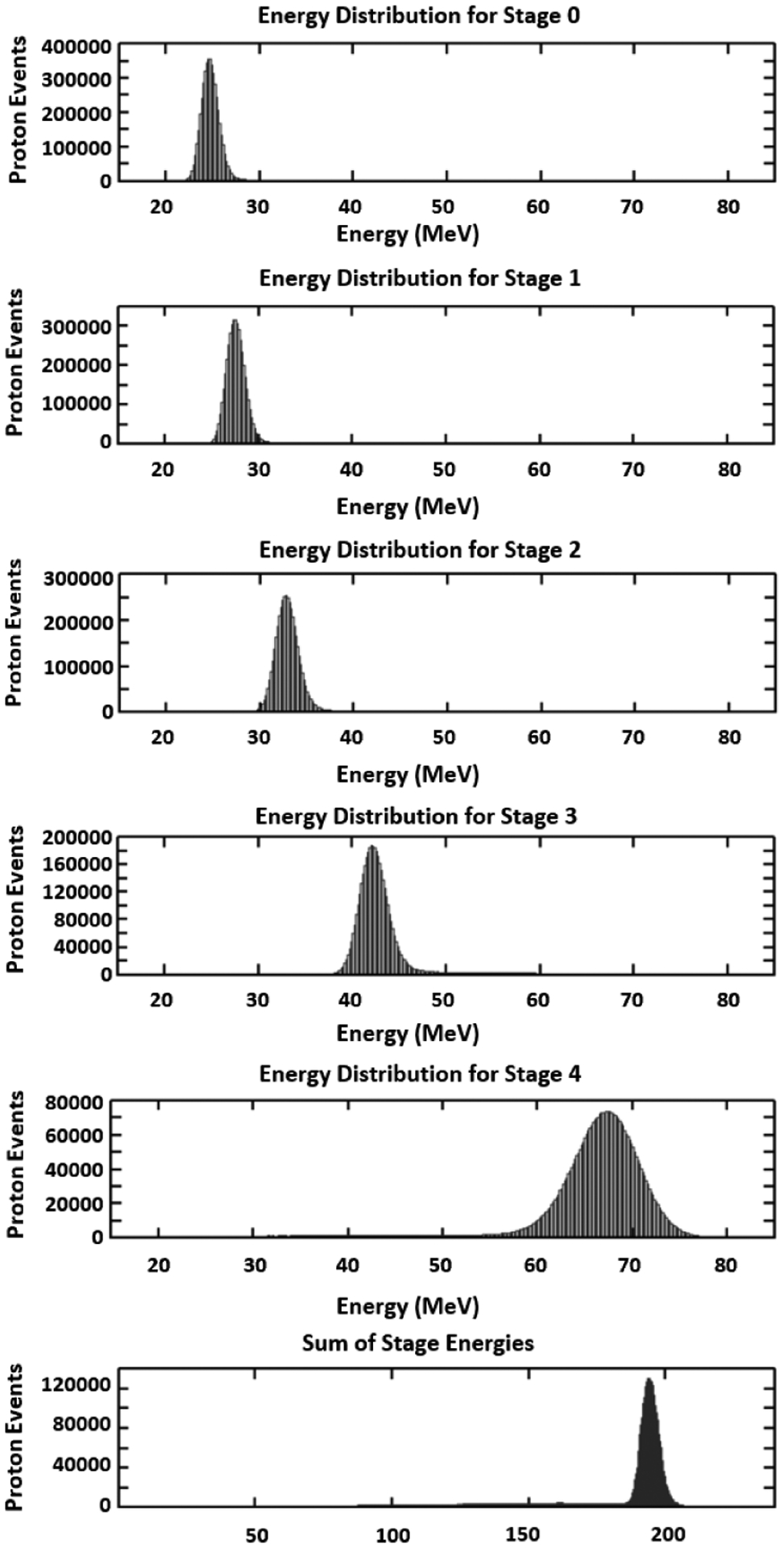
Energy deposited in the 5 stages of the energy detector for 200 MeV incident protons. Note that, in this case, the protons are stopping in Stage 4. Energy deposited increases with each stage due to increased energy loss as the protons slow down. The last graph is the sum of the 5 stages.

In order to convert the ADC values to MeV of energy deposition, a Monte Carlo simulation of the calorimeter was performed with 200 MeV protons and no phantom. This provides the calculated proton energy loss in each stage needed to convert pulse height to MeV. The result of an experimental calibration run with no phantom in place is shown in Fig. 3. The pulse heights have been converted to MeV for each stage using the calculated energy loss from this Monte Carlo simulation. The last graph in Fig. 3 shows the sum of the energies deposited in all stages, which adds up to about 195 MeV due to energy loss in the tracking detectors and other materials included in the beam delivery system.

**FIGURE 4.**
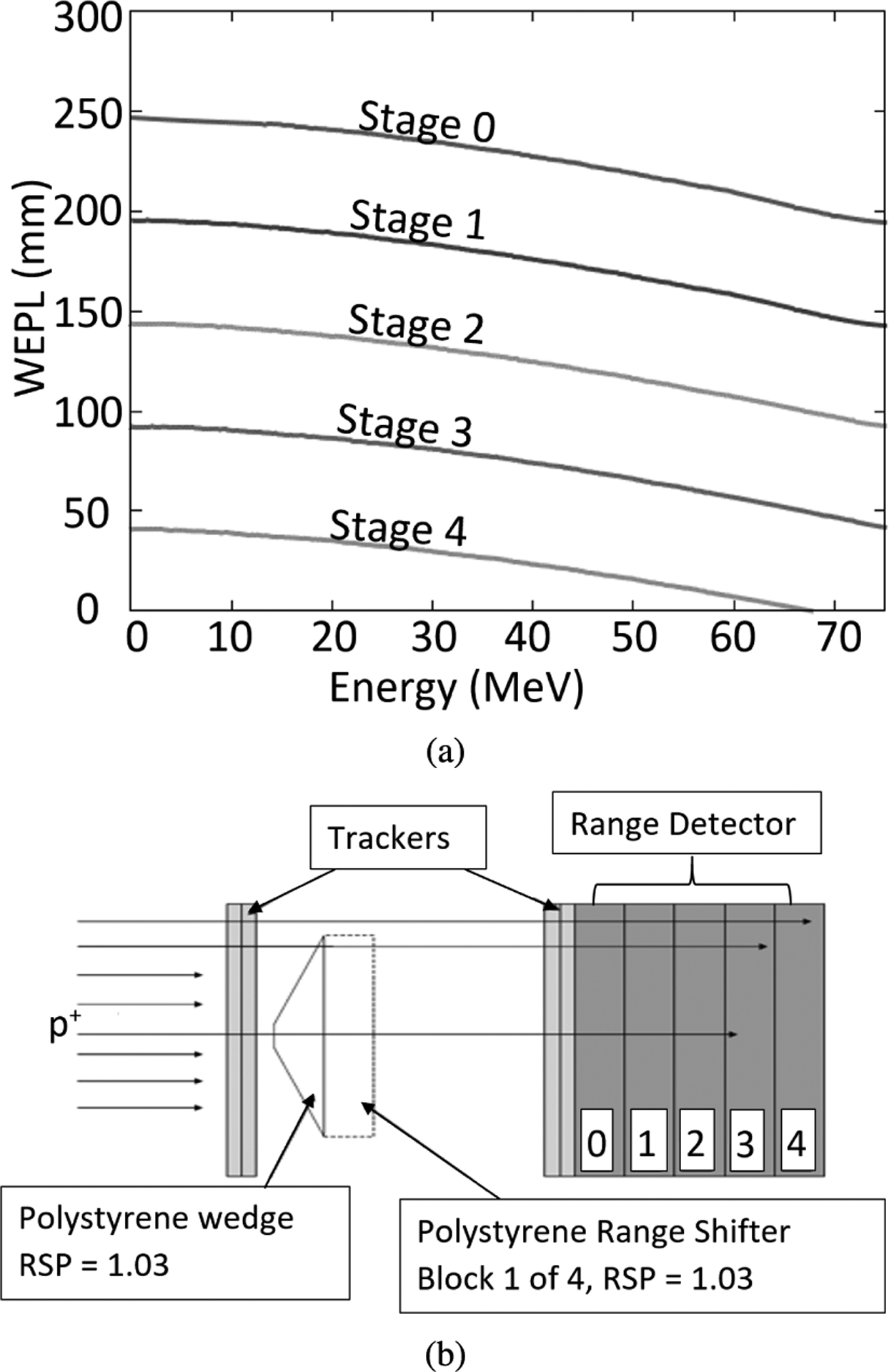
(a) Phantom WEPL vs. energy deposited in the scintillation stage where the protons stopped. (b) Wedge shaped calibration phantom with space for 4 insertable range shifter blocks (only one is shown).

In order to perform the calibration from pulse height (now expressed in MeV) to a WEPL value, several runs with 200 MeV protons are taken with a wedge phantom and zero to four additional polystyrene blocks (Fig. 4). The water equivalent path length (WEPL) for each track through the wedge depends on the entrance position as well as its trajectory. The maximum water equivalent thickness (WET) of the wedge, 52.34 mm, is close to the WET of the individual scintillator stages, 52.9 mm. The wedge produces a continuous range of position- and angle-dependent WEPL values for the incident 200 MeV protons. The tracker data are used to calculate the true WEPL as follows: A straight line trajectory calculated from the (*T, V*) planes to the front and rear surfaces of the calibration phantom defines the entrance and exit points of the path through the phantom. The physical path length is then calculated as the distance between these points and multiplied by the RSP of polystyrene (1.030) to obtain the WEPL. The WEPL is then correlated to the MeV value of the last stage with a signal in the range detector. For example, the Stage 4 curve in Fig. 4 (a) shows the calibration curve for just the wedge. The WEPL values for protons stopping in the other stages can be measured by adding between one and four 52.34 mm thick polystyrene blocks. The resulting five data sets are analyzed by calibration code that first applies thresholds to the measured energy depositions to find the stage in which the proton stopped. For each stage, those stopping protons are used to make a calibration table relating the proton energy to phantom WEPL as determined above.

## VI. DATA FORMAT AND PREPROCESSING

### A. DATA FORMAT AND DATA VOLUME

Each proton history is characterized by a rotation angle, a WEPL value, and four (*T, V*) tracker coordinate pairs; one coordinate pair from each of the four tracker planes, two in front and two behind the phantom (see Figure 1). The four coordinate pairs are then used to determine the angle of the proton as it enters and exits the phantom, respectively. The (*T, V*) coordinate information allows one to calculate the locations and path lengths of the voxels which were traversed by each proton. The pCT detector is capable of acquiring data at rates of 1 million protons per second with more than 90% live time, i.e., number of recorded events divided by the number of triggers. The data size for each proton history is about 70 bytes and typically 360 million proton histories are recorded for a six minute scan. This covers a cylindrical reconstruction volume of 25 cm diameter by 9 cm length centered on the axis of rotation. For phantoms with height more than 9 cm, two or more sets of scan data are acquired. The image sets then have their CT slices stitched together after the reconstruction to get the full field of view.

### B. PREPROCESSING OF DATA

Preprocessing of the data is required to reject errant histories and provide isocentric track coordinates in each plane for each proton. A calibration procedure for the energy detector is also performed to convert pulse height information to WEPL, as described in Section V. In addition to rejection of errant histories, the raw data must be processed to reduce each proton history to a set of ten parameters used by the image reconstruction software. These are four *T* coordinates and four *V* coordinates on the tracker planes, the angle *θ* of the rotation stage supporting the phantom, and the WEPL of the track calculated from the five-stage energy detector. Most importantly, it is necessary to remove events with more than one proton incident upon the five-stage detector because that detector is incapable of separately measuring their WEPLs if they arrive too close in time, i.e., less than 100 ns apart. This is accomplished primarily by requiring exactly one track to be found in each of the *T* and *V* views; only a limited number of extra, unassociated hits are allowed in the tracker. Cuts on the five-stage detector pulse heights can also remove multi-proton events if the pulse heights are too large for a single particle.

The raw tracker data consists of bit-packed lists of clusters of adjacent strips hit, usually with only one or two strips hit per cluster. After unpacking the data, the coordinates are assigned at the centers of the clusters and translated to the (*T, U, V*) coordinate system according to alignment constants generated from optical microscopy surveys of each of the detector planes. Small offsets of each plane in *T* and *V* are determined from fitting straight line proton tracks to the coordinates measured for millions of straight-through 200 MeV protons.

The PMT signals of the five-stage detector are digitized at 65 MHz by 14-bit ADCs. The pulse height is then taken to be a sum of six of the ADC samples, including the peak sample, one sample before the peak, and four samples after the peak. That data reduction is done in the FPGA of the digitizer board in order to minimize the event size.

### C. DERIVING THE (T, V) COORDINATES FROM THE TRACKER DATA

The 3D track pattern recognition operates independently in the two views, (*T, U*) and (*U, V*). In each view, all possible track segments are found in both the front and rear trackers by pairing a *T* and *V* hit in two successive planes. It is important to note that one cannot simply ignore proton histories with a missing *T* or *V* coordinate because they intersected one of the tracker gaps. It has been our experience that this leads to systematic image artifacts. This can be done accurately as follows. If there is no recorded *T* coordinate hit in either the front or rear tracker, then the centers of the gaps between sensors (which are parallel to the *T* strips) are considered as possible hit candidates. If there is still no *T* or *V* coordinate recorded in the front tracker, then the effective source points are used together with the recorded hit to generate the track segment. If there is only one hit recorded in the *V* coordinate of the rear tracker, then a rear track segment is generated by extrapolating the front tracker segment. Tracks are then kept only if the angle of each segment with respect to the beam direction does not exceed a specified limit, which is much tighter for the front set of trackers than the rear tracking planes due to scattering through the phantom. At this point, a complete set of coordinates (*T, V*) from the eight tracker planes are formed, which defines unique track segments upstream and downstream of the phantom.

### D. PMT AND PEDESTAL DRIFT CORRECTIONS

During a pCT scan with a phantom in place, the PMT gains and pedestals may drift. To correct for this, the preprocessing program accumulates histograms of the pulse height in each stage of the energy detector for just those protons that completely missed the phantom on either side. The peak location in each histogram is then used to renormalize the energy calibration per stage. Pedestal values are also tracked and corrected during the preprocessing.

### E. DATA REJECTION

The next step in preprocessing is to remove proton histories that do not contribute meaningful data, for example because the event underwent nuclear interactions. Table 1 summarizes the results of a data analysis taken from a 30-second calibration run. Tracker events are rejected if the projections of the upstream and downstream track segments onto the (*T, V*) plane at *U* = 0 miss each other by more than ±3 mm. These ±3 mm cuts are mostly done to remove proton histories that underwent nuclear interactions in the tracker planes or object and would not contribute meaningful data. Initially, triggered tracker events are accepted if they contain at least one good track. A good track is defined as a combination of tracker segments in each view that, when projected onto the central (*T, V*) plane (*U* = 0) are within ±3 mm of each other. From these events, those that have additional track segments or additional complete tracks will be rejected. For each proton surviving the tracker acceptance steps, the preprocessing applies the same thresholds as used in the calibration to determine in which stage it stopped. If the pulse height in the stopping stage is too large, the event is rejected, eliminating most multi-proton events missed by the tracker. If the pulse height in the preceding stages is below the range of the values expected for a complete proton Bragg peak curve, the event is rejected.

Overall, approximately 70% of the raw data events yield proton histories that serve as input to the image reconstruction program (Table 1). The two most important steps responsible for ~25% of data rejection are due to incomplete tracks and multiple track events. The remaining data loss (~5%) is due to pile-up events missed by the tracker data rejection steps and events inconsistent with the Bragg peak curve. Removing the errant events during the preprocessing phase has the added benefit of reducing the volume of data that needs to be transferred to/from and stored on the computation cluster performing the preconditioning steps, yielding a reduction in resource strain and reconstruction time.

The most common reason for data rejection at the tracker data analysis level is nuclear interactions in the object or tracker planes. These events often result in large-angle scattering of the protons, which then miss one or both of the down-stream tracker planes. The second most common reason is the arrival of two or (rarely) more protons during the tracking data acquisition window of 100 ns, leading to a pile-up rate of less than 10% at the operating proton rate of 10^6^ per second. Less frequent reasons for data rejection at the tracker level include noise hits and tracker inefficiency (<1%).

The most common reasons for data loss related to the pulse height analysis of the energy detector stages include pile-up of two or more protons in the energy detector, tracks leaving the detector before stopping, or inelastic nuclear interactions in the energy detector with the production of secondary neutral or charged particles.

Using 12 threads on an Intel(R) Xeon(R) E5–2695 v4 CPU at 2.10 GHz, the entire preprocessing (the first row of Table 2) of 360 million events, including statistical cuts, takes 6 minutes and 40 seconds. The proton histories are then ready for preconditioning.

## VII. PRECONDITIONING BEFORE ITERATIVE IMAGE RECONSTRUCTION

Preconditioning is a series of steps applied to the preprocessed data that leads to the initial solution serving as input to the iterative image reconstruction. In this article, we only discuss data acquisition, preprocessing and preconditioning. The iterative image reconstruction will be described in detail in a forthcoming companion paper. All steps following data acquisition are shown in Table 2.

In the following, we first describe a procedure to bin the WEPL data in a suitable format for a filtered backprojection (FBP) method based on a cone beam geometry that gives the initial RSP solution for the iterative image reconstruction.

### A. BINNING THE DATA FOR STATISTICAL CUTS AND FILTERED BACKPROJECTION

**FIGURE 5.**
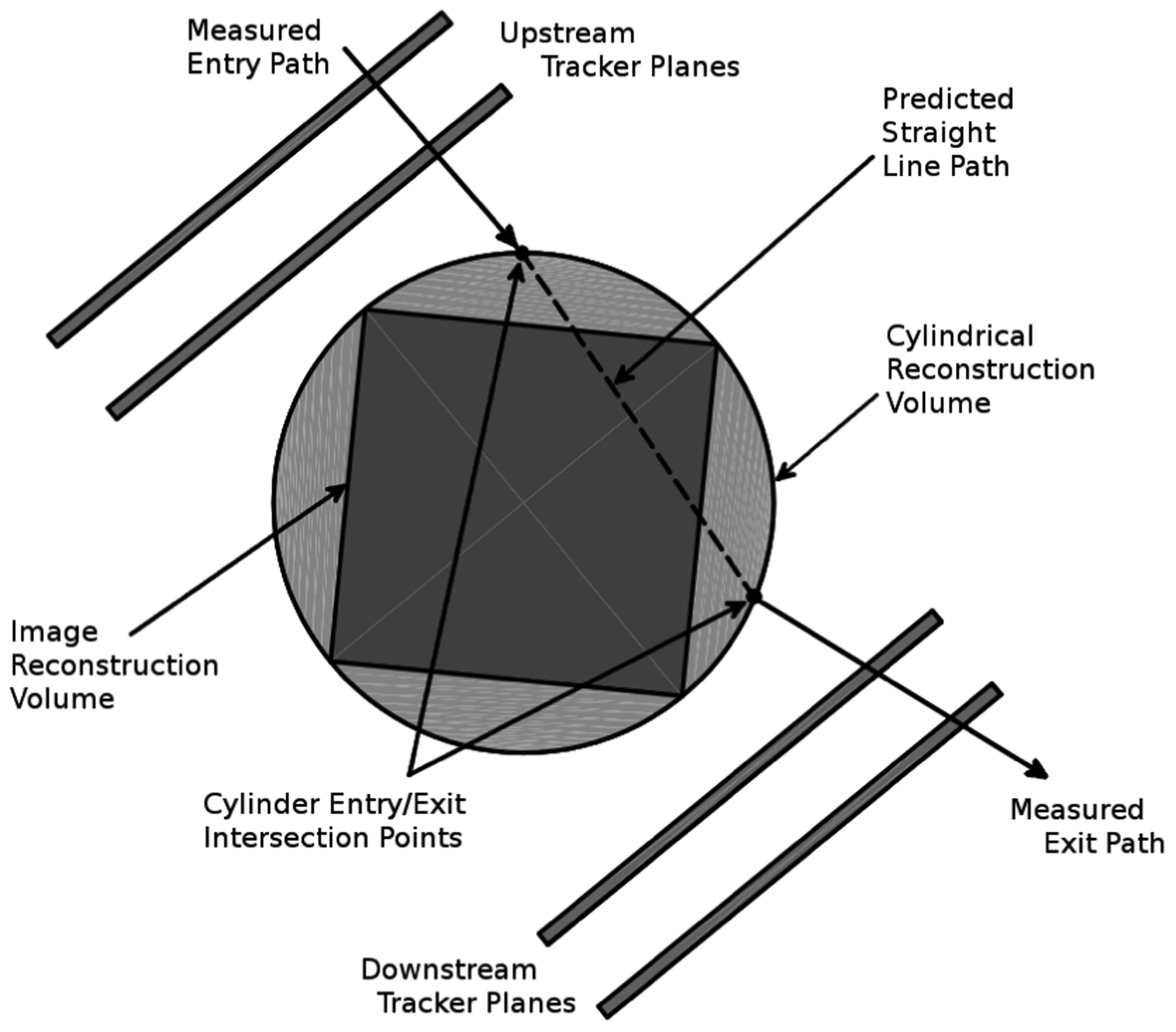
Forward- and backward-projection of the track segments onto the cylindrical reconstruction volume followed by a calculation of the predicted straight line path shown in the figure. The midplane for calculating FDK coordinates (*T*′*, V*′) lies parallel to the detector planes and centered on the rotation axis.

The beam delivery system has a divergent geometry produced by the scanning magnets. Therefore, we use the modified FBP algorithm established by Feldkamp, Davis, Kress (FDK algorithm) [21]. When the events are read into the reconstruction program, each hit on each tracker plane has its position calculated in the (*T, U, V*) coordinate system, as shown in Fig. 1. The maximum reconstruction volume is a 25 cm diameter by 9 cm length cylinder centered on the axis of rotation. First, a rotational transformation is applied to the measured hit locations such that the reconstruction can be carried out in the phantom-centered coordinate system (*X, Y, Z*). Next, each proton history is forward- and back-projected along the measured entry and exit directions, respectively, onto the surface of the reconstruction volume, as shown in Fig. 5.

**FIGURE 6.**
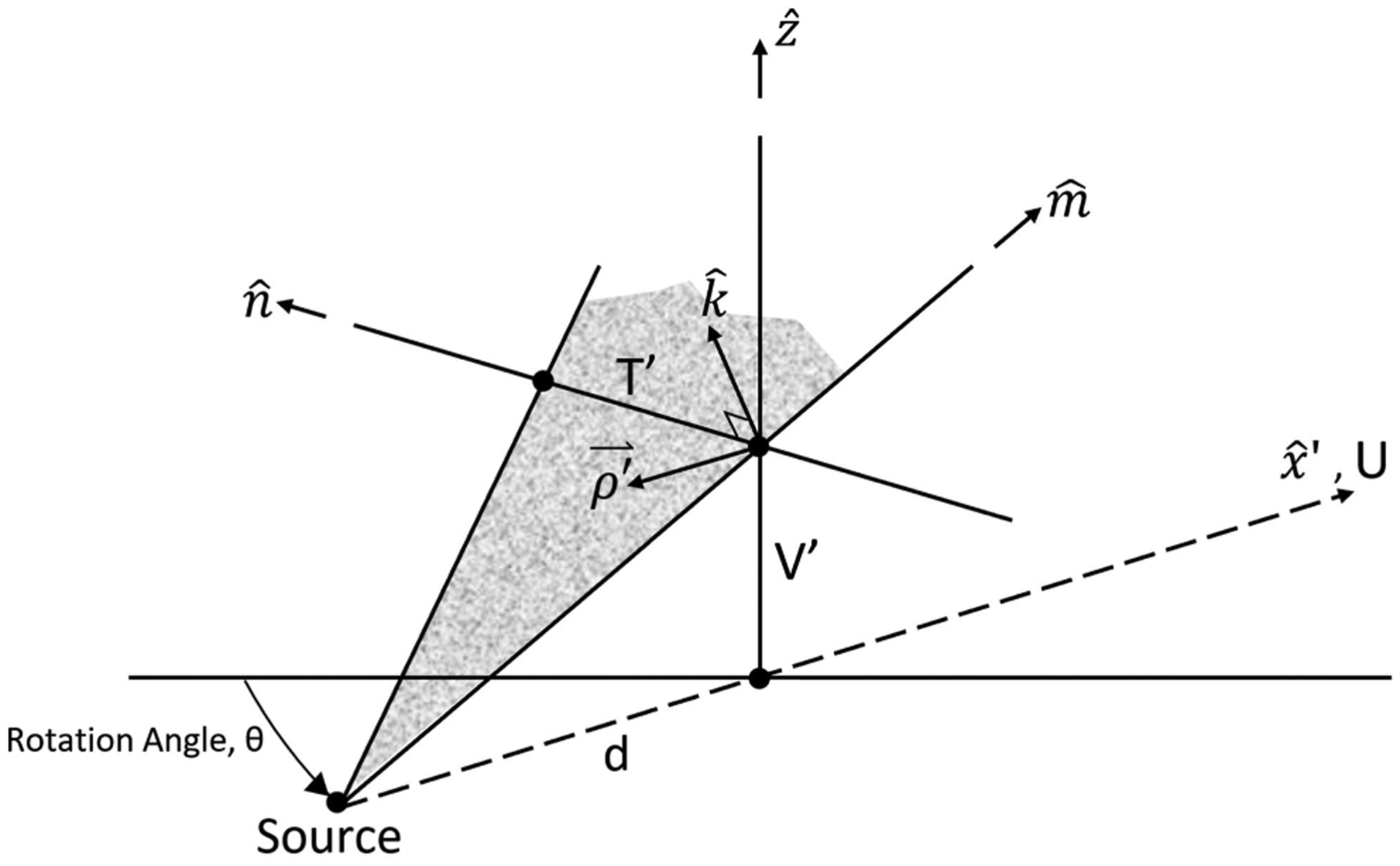
The coordinate system of the FDK algorithm for cone beam geometry modified from [21]. The axes and coordinate system from the pCT setup (Fig. 1) were added for clarity. The binning of data occurs in the (*T*′*, V*′) midplane, which is perpendicular to the $\hat{x}\prime$ axis (i.e., the *U* axis shown in Fig. 1). Original figure reproduced with permission from the Journal of the Optical Society of America.

A straight line approximation of the proton path through the reconstruction volume is made by joining the entry and exit points of the cylindrical reconstruction volume. This line segment has an angle *θ* and crosses the midplane at *U* = 0 with coordinates (*T*′*, V*′). The (*T*′*, V*′) coordinate system used in the FDK reconstruction is described in Fig. 6.

The distance from the source to isocenter, *d*, is set to 210 cm, the average of 190 cm and 230 cm, as defined by the distances of the *T* and *V* scanning magnets from isocenter. This is a pragmatic choice because, for distances of the source points from the isocenter (~200 cm) being large compared to the height of the reconstruction cylinder (9 cm), the effect of varying d by ± 20 cm on the quality of the FDK reconstructed images is small and further reduced during the final iterative image reconstruction. Proton histories are then binned according to their paths through the cylindrical reconstruction volume, with each proton history assigned to the (*T*′*, V*′*, θ*) bin through which the line segment crossed the midplane. Angular bin size, *δθ*, must be an integer divisor of 360° and is typically chosen to be 4°; *T*′ and *V*′ bin sizes are typically chosen to be *δT*′ = *δV*′ = 1 mm. We have also tried finer binning between 1° and 4° degrees, and while this improves the spatial resolution of the FDK reconstruction, it comes at the cost of increased noise due to the smaller number of protons registered in the smaller bins and additional problems with the straight-line path approximation. We continue to use 4 degrees as our standard bin size, but longer scans with a larger number of proton histories can also be used with smaller angular binning intervals for statistical cuts and FDK reconstruction. Each (*T*′*, V*′*, θ*) bin then contains proton histories that traversed similar paths through the cylindrical reconstruction volume, providing the means to perform a statistical analysis of the histories in each bin and remove outlier data.

### B. WEPL AND SCATTERING ANGLE DISTRIBUTIONS AND CUTS

A statistical analysis of the binned data is performed to identify and remove (cut) outlier proton histories, such as those associated with elastic and inelastic nuclear scattering and those that were not removed by the preprocessing rejection steps. These statistical cuts assume that a Gaussian approximation is valid for the central part of the actual distribution of scattering angles in T and V views and WEPL values converted from pulse height in the stopping stage of the energy detector. The benefit of these statistical cuts is that it improves RSP accuracy and spatial resolution. Generally, the underlying distributions of the WEPL and scattering angles are non-Gaussian and exhibit skew and positive kurtosis due to the combination of Gaussian- and non-Gaussian-distributed physical processes. It turns out that these effects are mostly limited to large-angle scattering events and large energy losses due to nuclear interactions of the protons. We have found that both distributions are sufficiently close to a Gaussian within 2*σ* (2 rms) of the mean. Therefore, the first choice is using 2*σ* cuts on the WEPL and scattering angle distributions to remove events due to infrequent physical processes. It is also possible to use the 3*σ* cuts suggested in earlier publications related to the pCT project [8], [22]. The difference between the two choices is relatively minor and is presented and discussed in the companion paper on image reconstruction.

**FIGURE 7.**
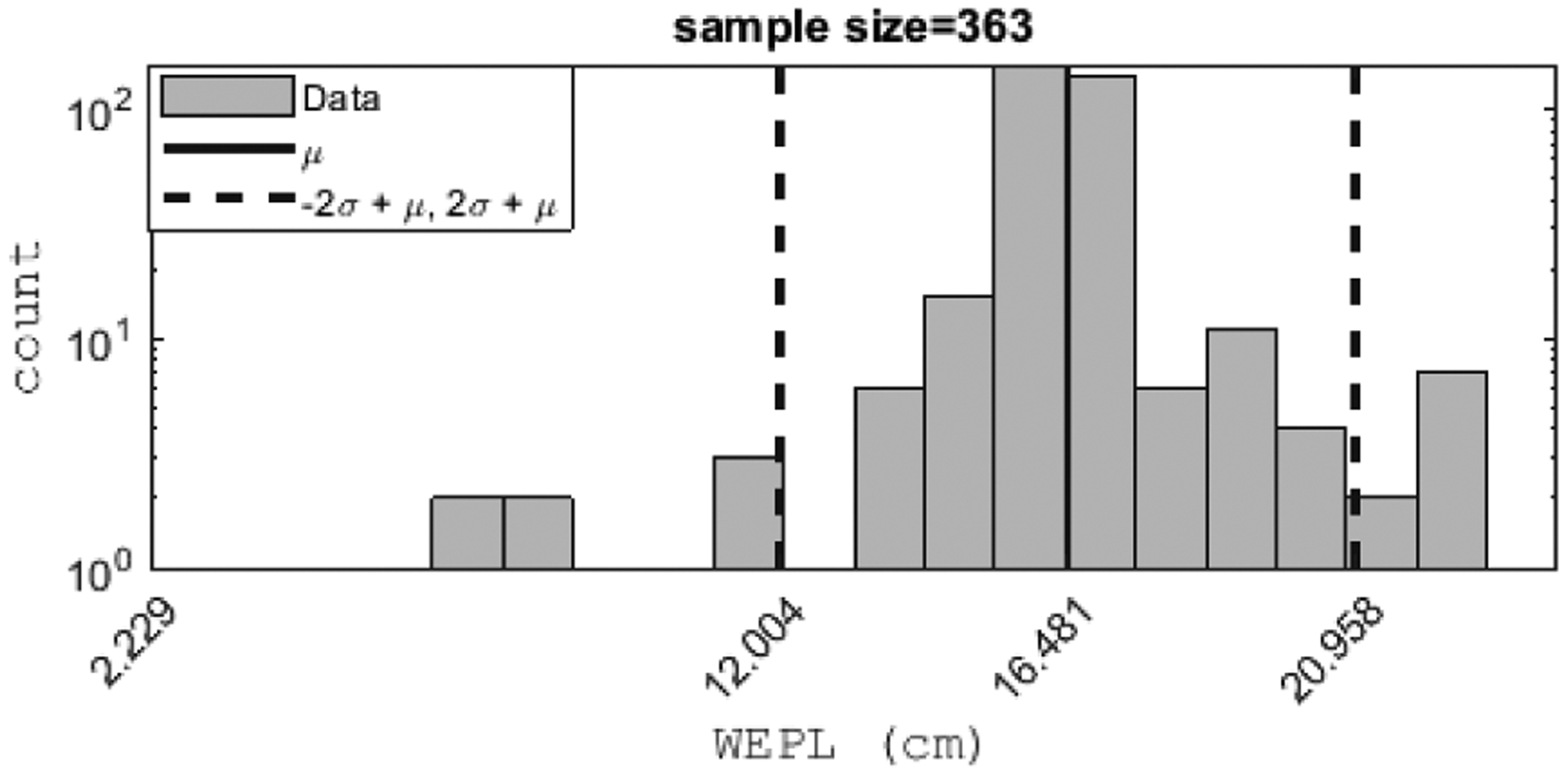
A typical WEPL distribution in a (*T*′*, V*′) bin near the center of the 15 cm diameter CTP404 phantom (shown in Fig. 8). The dashed line corresponds to 2*σ* WEPL cuts.

The mean WEPL value and the *σ* of each (*T*′*, V*′*, θ*) bin are then calculated and all proton histories whose WEPL deviates from the mean of that bin by more than the specified limit are cut. Fig. 7 shows an example that demonstrates the typical shape of the WEPL distribution and shows the location of 2*σ* WEPL cuts for a representative (*T*′*, V*′*, θ*) bin.

**FIGURE 9.**
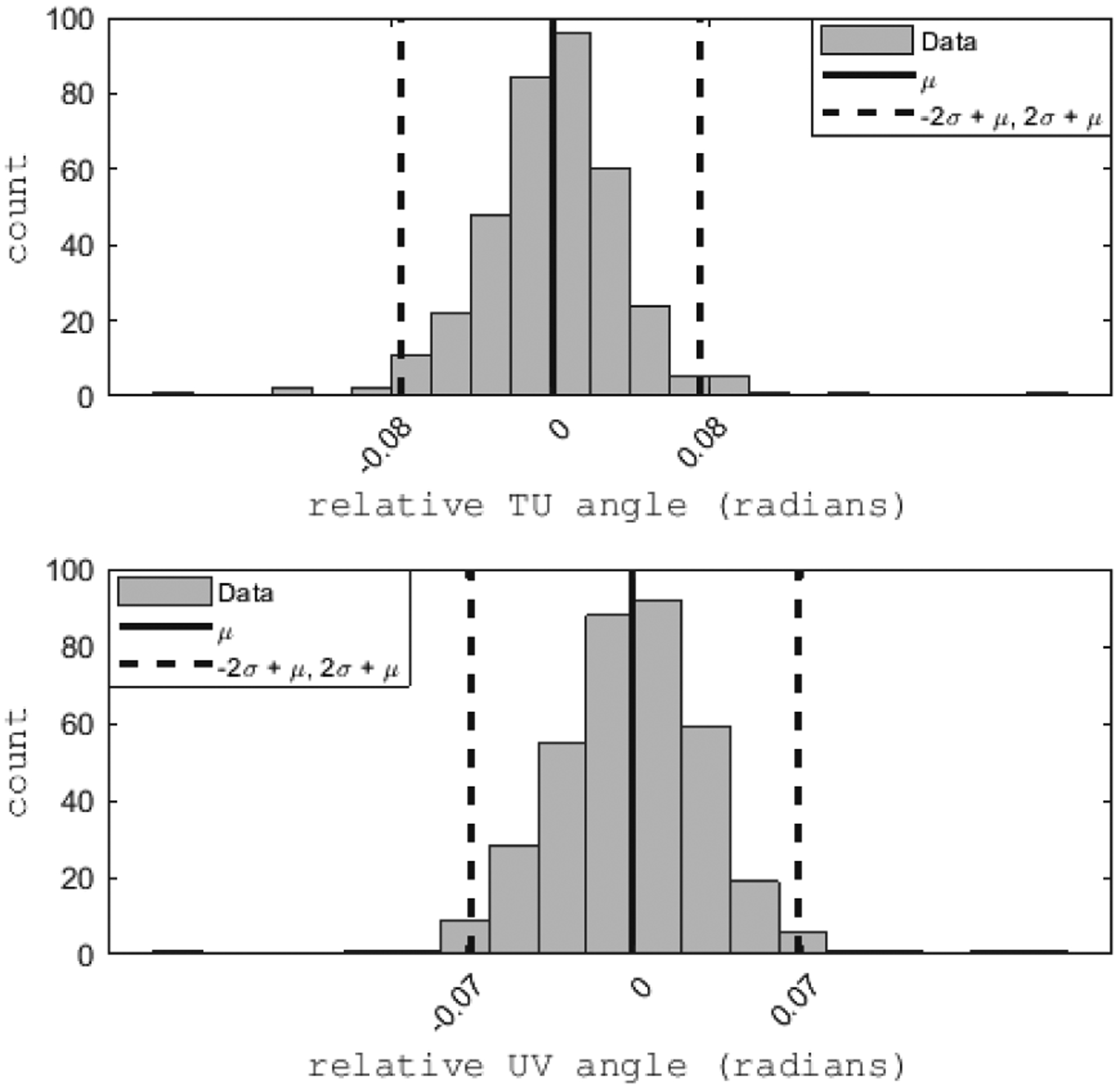
Distributions of the scattering angle between the upstream and downstream proton segments scored in a given bin of the *T*′ *V*′ midplane and a given angular bin *θ*. The top figure shows the distribution of 3D scattering angles projected onto the *TU* plane, and the bottom figure the distribution of 3D scattering angles projected onto the *UV* plane. The dashed line corresponds to 2*σ* scattering angle cuts. The sample size is 363.

Cuts are also performed based on scattering angle to remove large angle and other outlier scattering events, with the scattering in the *TU*− and *UV*−planes considered separately. For each (*T*′*, V*′*, θ*) bin, the scattering angles are calculated by taking the difference between the entrance and exit angles of each proton in both the *TU*− and the *UV*−planes. The mean and *σ* of the *TU*− and *UV*−scattering angles are then calculated and all proton histories whose *TU*− or *UV*−scattering angle deviates by more than a specified limit from their respective mean are removed. A typical example of a distribution of scattering angles with 2*σ* cuts is shown in Fig. 9.

### C. SINOGRAM

The proton histories remaining after WEPL and scattering angle cuts are subsequently used to generate the sinogram for the FDK reconstruction. A WEPL histogram is calculated from the WEPL values of each (*T*′*, V*′*, θ*) bin. For a typical object diameter with WEPL values up to 22 cm and more than 108 proton histories handled during preconditioning, the WEPL bin size is 5 mm. It can be larger, e.g., 10 mm, if the number of protons per (*T*′*, V*′*, θ*) bin is relatively small (see Fig. 7). The resulting histogram distribution is typically single-peaked (unimodal), but occasionally it has two distinct peaks (bimodal) when materials with two very different RSP values were present across the bin. For a uni-modal distribution, the mode is chosen as the representative WEPL value assigned to the (*T*′*, V*′*, θ*) bin. In contrast, it is calculated as the weighted mean of the two peak values for a bimodal distribution.

**FIGURE 10.**
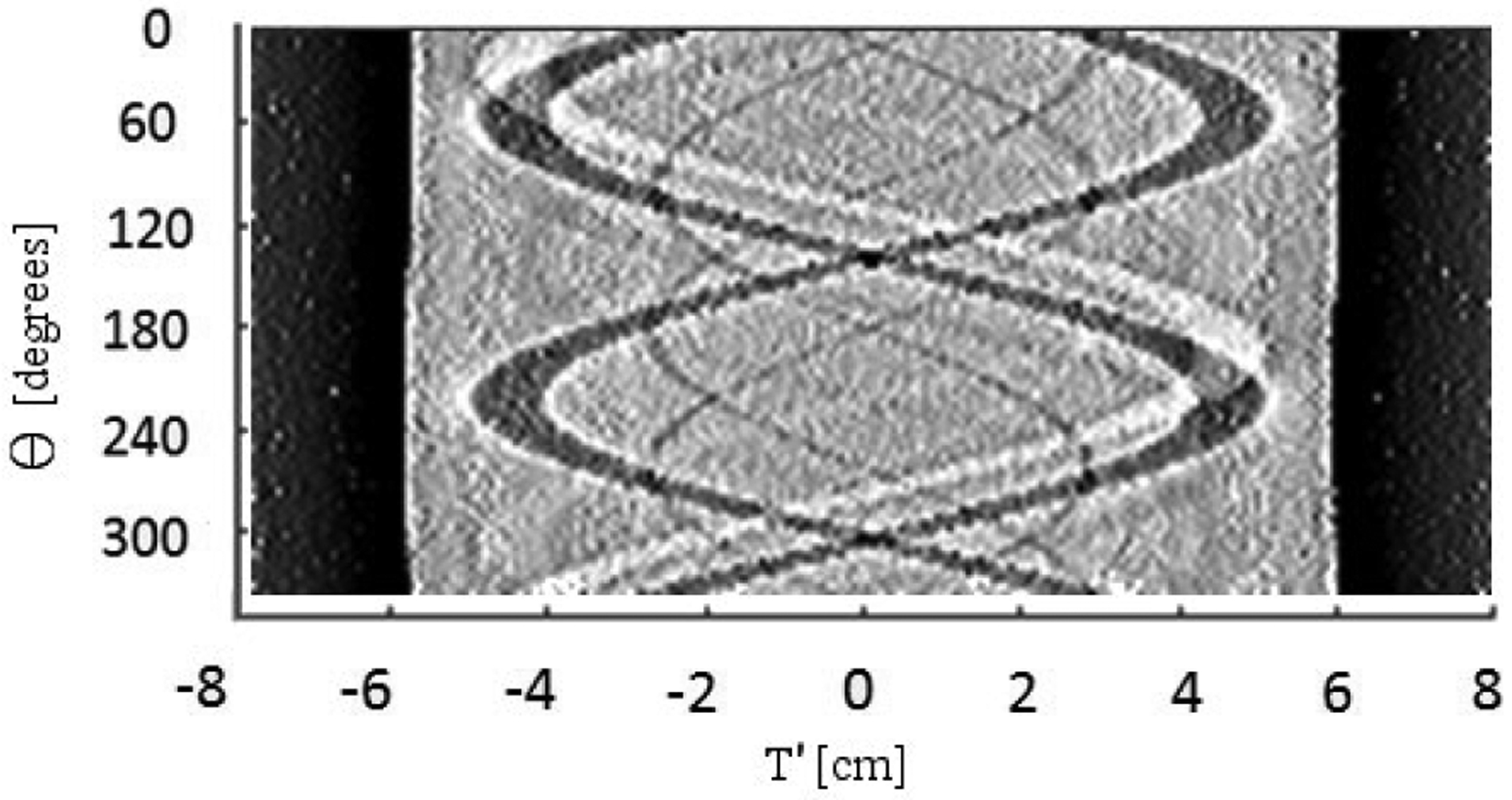
Filtered sinogram of experimental data for a single 1 mm thick axial slice through the middle of the 15 cm diameter CTP404 phantom shown in Fig. 8. This is a grayscale image of WEPL′(*T*′*, θ*). A Shepp-Logan filter, described in Section VII-D, was applied to the WEPL (*T*′*, θ*) sinogram data. The sinogram was generated with 350 bins for *T*′ and 90 bins for *θ*.

Once each (*T*′*, V*′*, θ*) bin has been assigned a representative WEPL value, i.e., the sinogram has been constructed, the Shepp-Logan filter (Equation 7) is applied to the WEPL data to generate the WEPL′ sinogram. Fig. 10 shows the central *V*′ slice of a representative WEPL′ sinogram derived from data of the CTP404 phantom. The horizontal axis displays the *T*′ coordinate varying from −80 mm to 80 mm, and the vertical axis varies from 0 to 360°.

### D. FDK METHOD OF FILTERED BACK PROJECTION

The equation to calculate RSP(*X, Y, Z*) = RSP(**r**) from the projection data, WEPL(*T*′*, V*′*, θ*) in the FDK algorithm, is given by

(4) $$\text{RSP}\left(r\right)=\frac{1}{4\pi^{2}}\int_{0}^{2\pi }\frac{d^{2}}{{\left(d+r\cdot \hat{x}\prime \right)}^{2}}\text{WEPL}\prime \left(T^{\prime }\left(r\right),V^{\prime }\left(r\right),\theta \right)d\theta$$

where *d* is the source to rotation axis distance (or SAD), $\hat{x}\prime$ is the unit vector perpendicular to the (*T*′*, V*′) plane (see Fig. 6) and WEPL′ is the Shepp-Logan filtering of WEPL(*T*′*, V*′*, θ*). Also, *T*′ and *V*′ are parameterized in terms of the vector **r**, as follows:

(5) $$T^{\prime }\left(r\right)=r\cdot \hat{n}\frac{d}{d+r\cdot {\hat{x}}^{\prime }}$$

(6) $$V^{\prime }\left(r\right)=r\cdot \hat{z}\frac{d}{d+r\cdot {\hat{x}}^{\prime }}$$

The equations for WEPL′ are adapted from the FDK equations (31) - (33) [21], but are given here for completeness, i.e.,

(7) $$\text{WEPL}\prime \left(T^{\prime },V^{\prime }\right)=\int_{-\infty }^{+\infty }\int_{-\infty }^{+\infty }\text{WEPL}\left(T,V\right)g_{1}\left(T^{\prime }-T\right)g_{2}\left(V^{\prime }-V\right)\times \frac{d}{\sqrt{d^{2}+T\prime^{2}+V\prime^{2}}}dT\;dV$$

and

(8) $$g_{1}\left(Q\right)=\text{Re}\int_{0}^{\omega_{T_{0}}}\omega \text{ exp}\left(i\omega Q\right)d\omega$$

(9) $$g_{2}\left(P\right)=\frac{\text{sin}\left(\omega_{V_{0}}P\right)}{P\pi }$$

The maximum spatial frequencies are given by $\omega_{T_{0}}$ and $\omega_{V_{0}}$. These are based on the finite spatial resolution determined by the pitch of the silicon strips, 0.228 mm, of the *T* and *V* tracker planes. With these values in the FDK equations, $\omega_{T_{0}}=\omega_{V_{0}}=\frac{\pi }{0.228\text{mm}}$. The RSP(**r**) can then be calculated from Equation 4, which gives the initial reconstructed image for input to the subsequent iterative image reconstruction.

**FIGURE 11.**
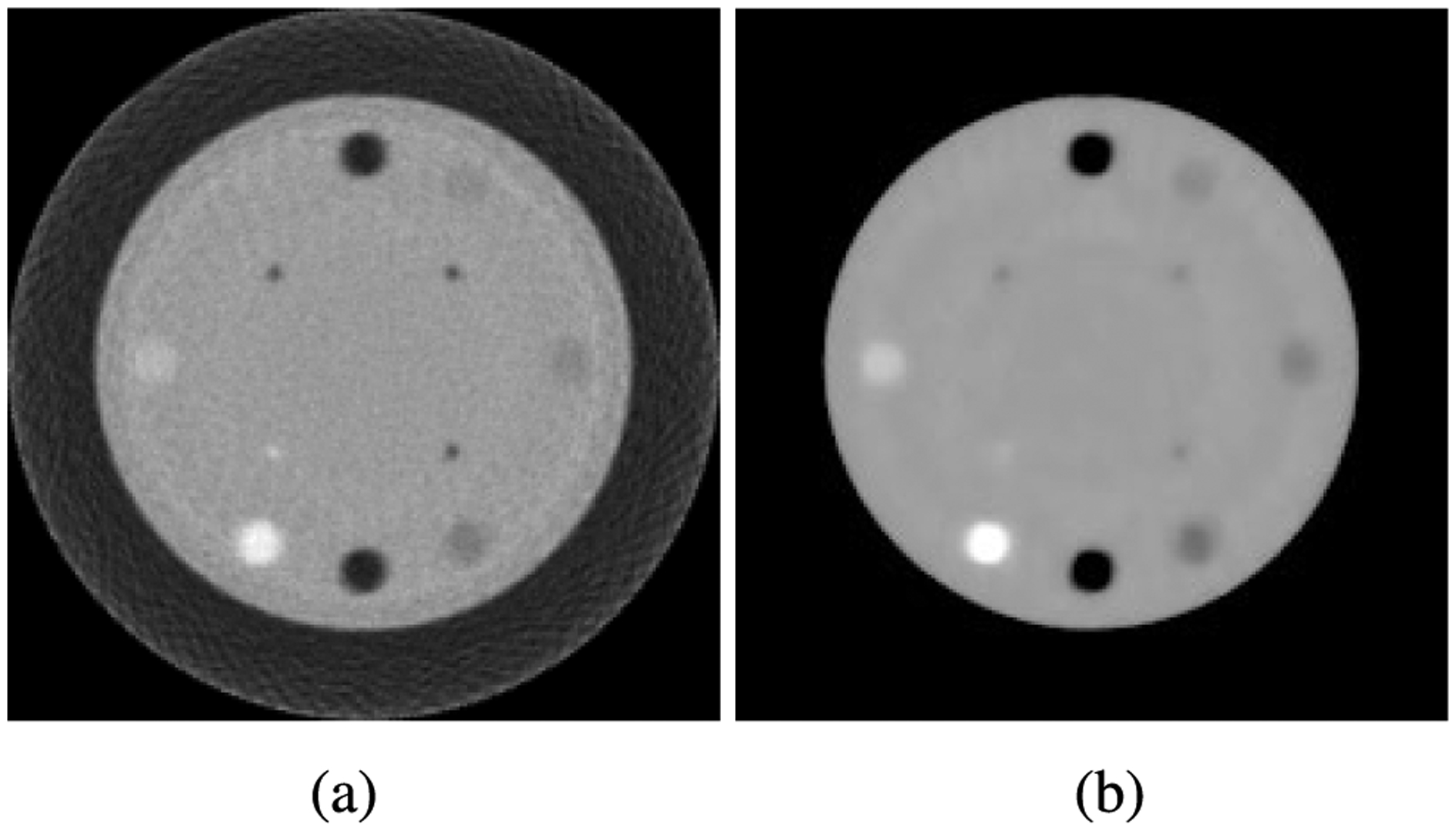
(a) An example of the FDK reconstructed image using the experimental CTP404 phantom with 2*σ* WEPL and scattering cuts. (b) Result of applying a median filter with a 3 voxel radius to the FDK image in (a) and then setting the voxels outside the phantom boundary to zero.

Applying these equations to the sinogram data for the CTP404 phantom gives the initial reconstructed image. The central slice (*Z* = 0) of the FDK reconstruction of RSP(*X, Y, Z*) is shown in Fig. 11(a). It should be noted that the image quality deteriorates slightly as the vertical distance from the central slice increases. However, this does not affect the visual image quality and quantitative image accuracy of the final iteratively reconstructed images, as presented and discussed in the second part of this contribution. The 12 mm diameter inserts of various materials are clearly visible. Finally, we note that the total time required to generate the FDK image shown in Fig. 11, i.e. the first 5 steps of the image reconstruction section in Table 2, was 53 seconds on a single P100 GPU. This time is consistent with other data sets of comparable size.

### E. IMAGE PROCESSING FOR NOISE REDUCTION

The initial iterate generated by the FDK reconstruction is inherently noisy due to statistical variations, the discretization of proton histories, and making use of a straight-line assumption. The iterative image reconstruction algorithms cannot remove noise from the images; therefore, reducing noise in the FDK reconstruction is important. Both mean and median filters can be used for noise reduction. A crucial feature of median filtering is its ability to preserve edges, which is important for pCT reconstruction. For this reason, the median filter was chosen as the noise-reducing filter of the initial FDK reconstruction. Admittedly, the median filter will reduce fine details in the reconstructed images; however, these will be recovered during the subsequent image reconstruction. An example of the median filter applied to an unfiltered FDK reconstruction (Fig. 11(a)) is shown in Fig. 11(b). Of note, in addition to median filtering, an algorithm for hull detection, including setting the RSP values outside the hull to zero, was applied. Details of hull detection algorithms are described and discussed in the companion paper on image reconstruction.

### F. FDK IMAGES FOR A PEDIATRIC HEAD PHANTOM

**FIGURE 12.**
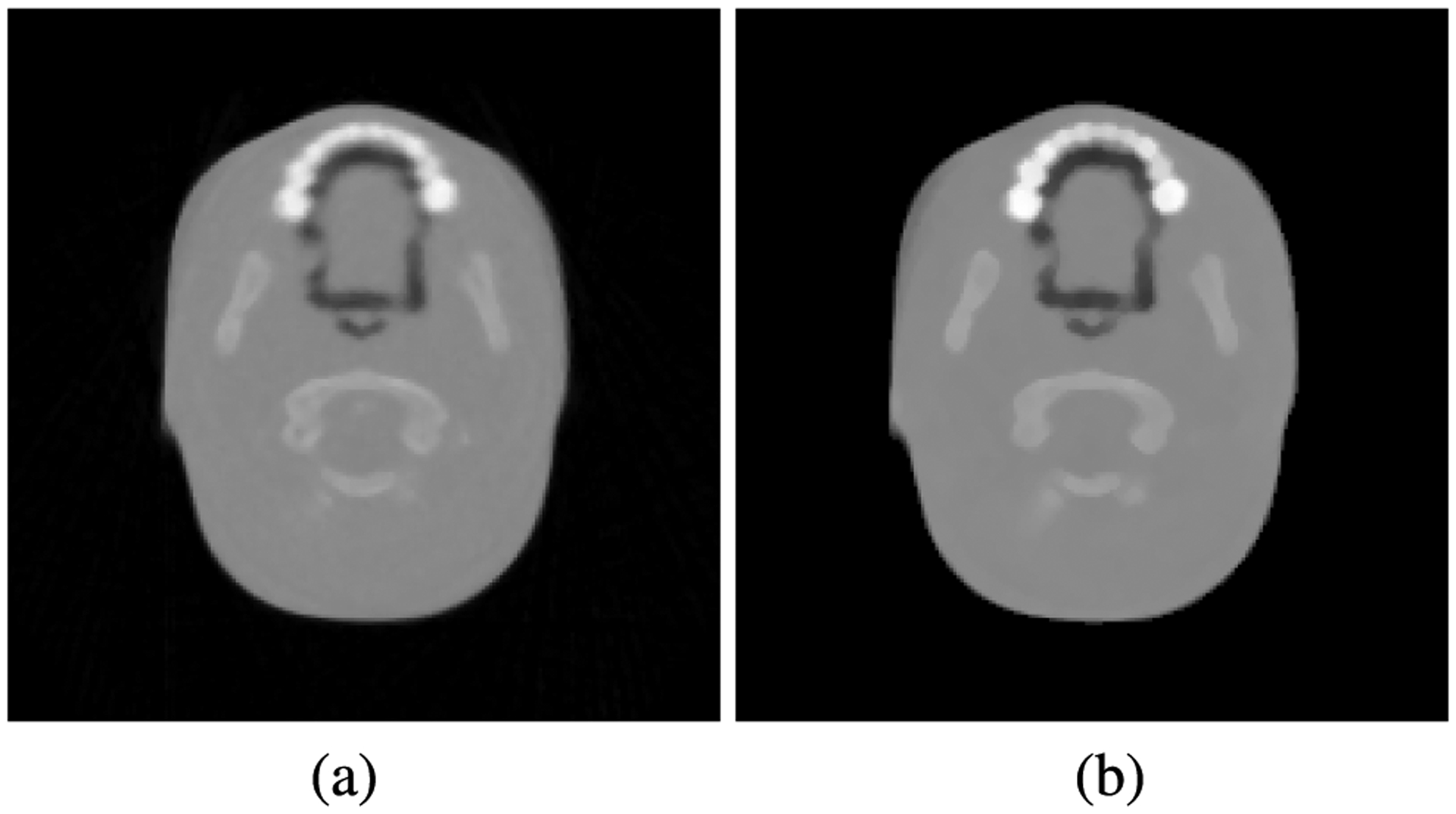
(a) An example of the FDK reconstruction of the pediatric head phantom using 2*σ* cuts on WEPL and scattering angles. A median filter with a 3 voxel radius was used on the FDK images. (b) Full iterative reconstruction using the FDK reconstruction as the initial iterate.

We have also scanned a pediatric head phantom (model HN715, CIRS, Norfolk, VA, USA). Note that because the detector has a limited field of view, 9 × 36 cm, the head phantom is scanned in two parts. A near-central slice (*Z* = 0) of a pCT reconstruction of the inferior portion of the head phantom can be seen in Fig. 12(a). The FDK reconstruction serves as the initial iterate for the subsequent iterative image reconstruction, resulting in Fig. 12(b). Commonly seen image degradation of the FDK image for off-central slices is not propagated into the final iterative image reconstruction, as discussed in the second part of this contribution.

These results show that the preprocessing and preconditioning techniques give a reasonable starting point for iterative image reconstruction. In a forthcoming paper, we will discuss RSP accuracy and spatial resolution in detail when iterative reconstruction techniques and MLP formalism are implemented to improve image quality. For example, we will demonstrate that RSP accuracy can generally be improved to better than 1%, which exceeds that of x-ray CT, and spatial resolution in a head-sized object can be improved to better than 1 mm while still not reaching the high resolution of x-ray CT.

## VIII. SUMMARY AND CONCLUSION

As the interest in the various forms of pCT grows, it is important to establish the major design issues and trade-offs to make a functional system. In this article, we presented a unified, complete overview of the engineering choices we have found to be the best for our pCT system. The underlying theory and many of the key issues encountered in earlier works that will facilitate further development, verification, and adoption by other groups were addressed.

In Section II, we presented the historical development of pCT, starting with Allan Cormack’s pioneering work. The history of pCT is tightly connected with CT in general, and though there are obvious differences discussed in this article and elsewhere, the similarities will likely keep the close connection going for years to come.

In Section III, we covered the designs of the tracker and energy detector. Key aspects of the design centered on a) the ability to produce a low intensity beam from a typical proton delivery system, b) the need to calculate the WEPL (Section IV), and c) performing the associated WEPL calibration (Section V).

In Section VI, we discussed the formatting and preprocessing of the data, including transformation of tracker hit data to the isocenter coordinate system and the rejection of unsuitable events. The resulting preprocessed data undergoes a preconditioning step to provide an initial image based on the FDK algorithm, as described in Section VII. Protons that intersect the cylindrical reconstruction volume are identified and binned for FDK to generate the initial iterate. Angle and WEPL cuts are used to eliminate protons whose interactions would result in lower quality reconstructions. The resulting data and images are then passed onto the iterative reconstruction algorithm, which is the subject of a forthcoming paper.

## Figures and Tables

**FIGURE 8.**
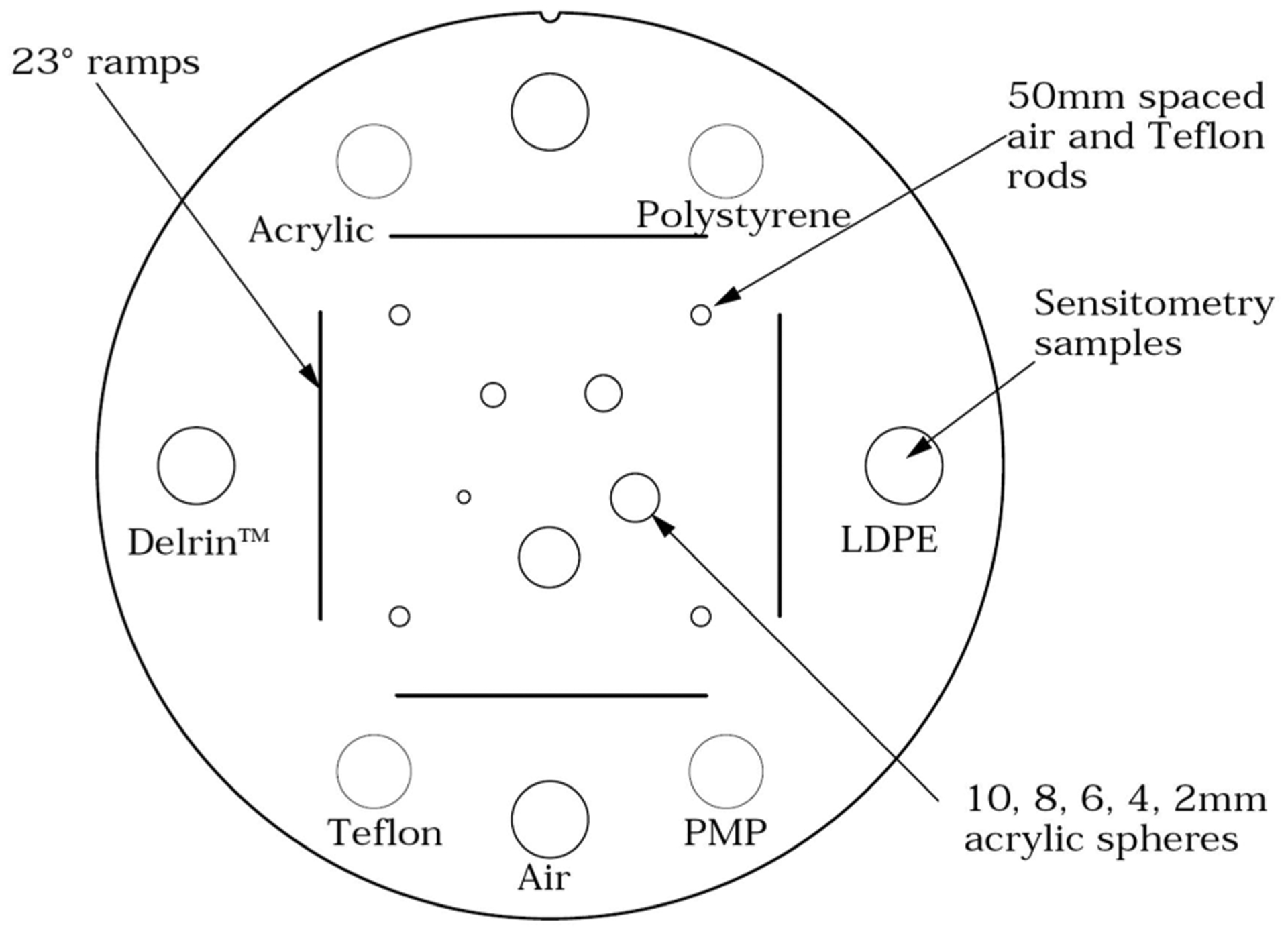
Diagram from the Catphan^®^ 500 and 600 Manual (The Phantom Laboratory Incorporated, Salem, NY, USA) demonstrating the CTP404 phantom.

**TABLE 1.** Representative Example Showing the Number of the Triggered DAQ Events (Absolute and Percentage) That Survived Data Rejection Steps During the Preprocessing Stage.

Acceptance Step	Number	Percentage
Trigger accepted	27,546,422	100%
At least 1 good track	23,310,729	84.6%
No evidence of a second proton in the tracker	20,319,265	73.8%
Stopping stage pulse height not too high	19,478,973	70.7%
Proceeding stages pulse heights not too low	18,960,588	68.8%

**TABLE 2.** Steps of Preprocessing and Image Reconstruction. The Last Two Steps of Image Reconstruction Will be Addressed in a Subsequent Paper.

Process	Step
Preprocessing	
	Read stored events from data acquisition computer Search data for “good” tracks and “good” energy detector signals Reduce each history to 4 (*T*, *V*) coordinate pairs, 1 WEPL, and 1 projection angle
Preconditioning	
	Calculate entry and exit points in isocentric coordinates on surface of cylindrical reconstruction volume for each track Perform FDK binning of data in (*T*′,*V*′) planes with straight line approximation Perform angular and WEPL cuts Create sinograms Perform FDK image reconstruction to get 1^1*st*^ RSP solution (initial iterate)
Iterative Image Reconstruction	
	Create object boundary definition, i.e., the hull, by applying an edge filter to 3D RSP images and tracks missing the phantom Create MLPs and iterative reconstruction of RSP
